# 7-Phenyl­sulfonyl-2,3-dihydro-7*H*-1,4-benzodioxino[6,7-*b*]carbazole

**DOI:** 10.1107/S1600536810047343

**Published:** 2010-11-20

**Authors:** J. Kanchanadevi, V. Dhayalan, A. K. Mohanakrishnan, G. Anbalagan, G. Chakkaravarthi, V. Manivannan

**Affiliations:** a Department of Physics, Velammal Institute of Technology, Panchetty, Chennai 601 204, India; b Department of Organic Chemistry, University of Madras, Guindy Campus, Chennai 600 025, India; c Department of Physics, Presidency College (Autonomous), Chennai 600 005, India; d Department of Physics, CPCL Polytechnic College, Chennai 600 068, India; e Department of Research and Development, PRIST University, Vallam, Thanjavur 613 403, Tamil Nadu, India

## Abstract

In the title compound, C_24_H_17_NO_4_S, the phenyl ring makes a dihedral angle of 88.12 (5)° with the carbazole unit. The mol­ecular structure is stabilized by weak intra­molecular C—H⋯O inter­actions and the crystal packing exhibits weak inter­molecular C—H⋯O and C—H⋯π inter­actions. Two C atoms of the 2,3-dihydro-1,4-dioxine fragment are disordered over two positions with site-occupancy factors of 0.718 (11) and 0.282 (11).

## Related literature

For the biological activity of carbazole derivatives, see: Ramsewak *et al.* (1999 ▶); Tachibana *et al.* (2001 ▶). For the structures of closely related compounds, see: Chakkaravarthi *et al.* (2008**a* ▶,b* ▶).
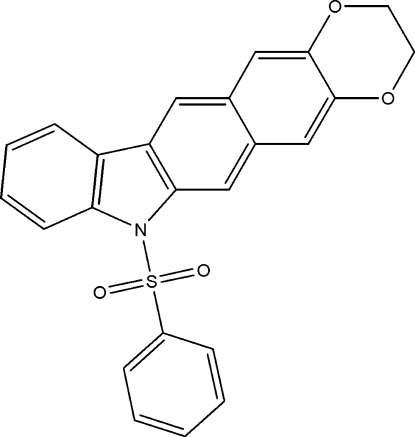


## Experimental

### 

#### Crystal data

C_24_H_17_NO_4_S
*M* *_r_* = 415.45
Orthorhombic, 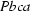

*a* = 13.189 (5) Å
*b* = 16.363 (6) Å
*c* = 18.039 (5) Å
*V* = 3893 (2) Å^3^
*Z* = 8
Mo *K*α radiation
μ = 0.20 mm^−1^
*T* = 295 K
0.26 × 0.22 × 0.20 mm

#### Data collection

Bruker Kappa APEXII diffractometer
Absorption correction: multi-scan (*SADABS*; Sheldrick, 1996 ▶) *T*_min_ = 0.950, *T*_max_ = 0.961
19551 measured reflections
4813 independent reflections
3469 reflections with *I* > 2σ(*I*)
*R*_int_ = 0.032

#### Refinement

*R*[*F* ^2^ > 2σ(*F* ^2^)] = 0.041
*wR*(*F* ^2^) = 0.124
*S* = 1.03
4813 reflections
290 parameters
1 restraint
H-atom parameters constrained
Δρ_max_ = 0.28 e Å^−3^
Δρ_min_ = −0.37 e Å^−3^

### 

Data collection: *APEX2* (Bruker, 2004 ▶); cell refinement: *SAINT* (Bruker, 2004 ▶); data reduction: *SAINT*; program(s) used to solve structure: *SHELXS97* (Sheldrick, 2008 ▶); program(s) used to refine structure: *SHELXL97* (Sheldrick, 2008 ▶); molecular graphics: *PLATON* (Spek, 2009 ▶); software used to prepare material for publication: *SHELXL97*.

## Supplementary Material

Crystal structure: contains datablocks I, global. DOI: 10.1107/S1600536810047343/gk2320sup1.cif

Structure factors: contains datablocks I. DOI: 10.1107/S1600536810047343/gk2320Isup2.hkl

Additional supplementary materials:  crystallographic information; 3D view; checkCIF report

## Figures and Tables

**Table 1:** Hydrogen-bond geometry (Å, °) *Cg*1, *Cg*4, *Cg*5 and *Cg*7 are the centroids of the N1/C7/C18/C19/C24, C1–C6, C7–C9/C16–C18 and C19–C24 rings, respectively.

*D*—H⋯*A*	*D*—H	H⋯*A*	*D*⋯*A*	*D*—H⋯*A*
C8—H8⋯O2	0.93	2.42	3.005 (2)	120
C23—H23⋯O1	0.93	2.32	2.908 (3)	121
C10—H10⋯O1^i^	0.93	2.52	3.411 (2)	161
C12—H12*A*⋯*Cg*1^ii^	0.97	2.98	3.670 (5)	129
C12—H12*B*⋯*Cg*7^ii^	0.97	2.78	3.411 (5)	124
C13—H13*A*⋯*Cg*5^iii^	0.97	2.77	3.680 (5)	157
C20—H20⋯*Cg*4^iv^	0.93	2.94	3.689 (2)	138
C12*A*—H12*C*⋯*Cg*5^iii^	0.97	2.53	3.446 (14)	158
C12*A*—H12*D*⋯*Cg*7^ii^	0.97	2.69	3.585 (14)	153
C13*A*—H13*D*⋯*Cg*1^ii^	0.97	2.92	3.585 (14)	127

Symmetry codes: (i) 
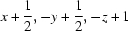
; (ii) 
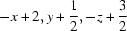
; (iii) 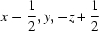; (iv) 
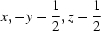
.

**Fig. 1.**
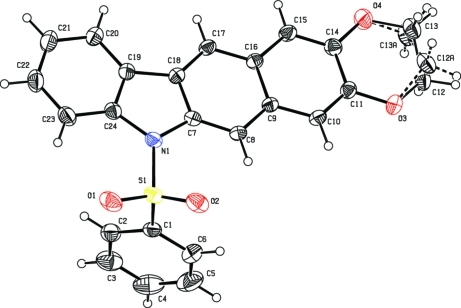
The molecular structure of the title compound with atomic labels and 30% probability displacement ellipsoids for non-H atoms.

**Fig. 2.**
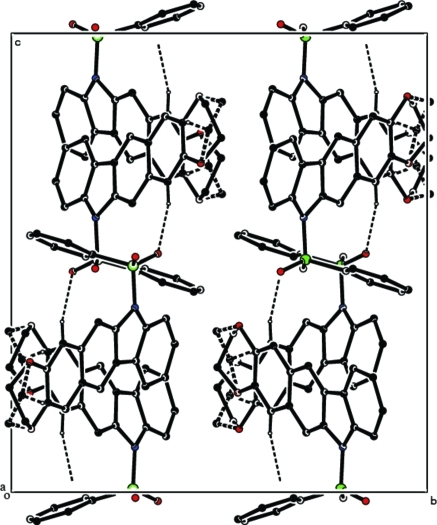
The crystal packing viewed down the a axis. Intermolecular hydrogen bonds are shown as dashed lines. H atoms not involved in hydrogen bonding have been omitted for clarity.

## supplementary crystallographic information

### Comment

Carbazole derivatives possess antioxidative (Tachibana *et al.*, 2001) and anti-inflammatory and antimutagenic (Ramsewak *et al.*, 1999) activities. The geometric parameters of the molecule (Fig. 1) agree well with the reported similar structures (Chakkaravarthi *et al.* 2008*a*,*b*).

The phenyl ring makes the dihedral angle of 88.12 (5)° with the carbazole ring system. The sum of the bond angles around N1 [357.29 (11)°] indicates that N1 atom is *sp*^2^ hybridized. The molecular structure is stabilized by weak intramolecular C—H···O interactions and the crystal packing exhibits weak intermolecular C—H···O (Fig. 2) and C—H···π interactions[C12—H12A···*Cg*1(2 - *x*, 1/2 + *y*, 3/2 - *z*) distance of 3.670 (5) Å; C12—H12B···*Cg*7 (2 - *x*, 1/2 + *y*, 3/2 - *z*) distance of 3.411 (5) Å; C13—H13A···*Cg*5 (1/2 + *x*, *y*, 3/2 - *z*) distance of 3.680 (5) Å; C20—H20··· *Cg*4 (*x*, 1/2 - *y*, 1/2 + *z*) distance of 3.689 (2) Å; C12A—H12C···*Cg*5 (1/2 + *x*, *y*, 3/2 - *z*) distance of 3.446 (14) Å; C12A—H12D···*Cg*7 (2 - *x*, 1/2 + *y*, 3/2 - *z*) distance of 3.585 (14) Å; C13A—H13D···*Cg*1 (2 - *x*, 1/2 + *y*, 3/2 - *z*) distance of 3.585 (14) Å; *Cg*1,*Cg*4,*Cg*5 and *Cg*7 are the centroids of the rings N1/C7/C18/C19/C24, C1—C6, C/7/C8/C9/C16/C17/C18 and C19—C24, respectively].

### Experimental

To a solution of diethyl-2-((2-(bromomethyl)-1-(phenylsulfonyl) -1*H*-indol-3-yl) methylene)malonate (0.5 g, 0.96 mmol) in dry DCE (15 ml), anhydrous ZnBr_2_ (0.43 g, 1.90 mmol) and 1,4-benzodioxane (0.14 ml, 1.17 mmol) were added. It was stirred at room temperature for 2 h and then refluxed for 0.5 h under N_2_ atmosphere. The solvent was removed and the residue was quenched with ice-water (50 ml) containing 1 ml of conc.HCl, extracted with chloroform (2 *x* 10 ml) and dried (Na_2_SO_4_). Removal of solvent followed by flash column chromatographic purification (n-hexane/ethyl acetate 99:1) led to the isolation of colourless crystal of the title compound.

### Refinement

H atoms were positioned geometrically and refined using riding model with C—H = 0.93 Å and *U*_iso_(H) = 1.2Ueq(C) for aromatic C—H and C—H = 0.97 Å and *U*_iso_(H) = 1.2Ueq(C) for CH_2_. The site occupancy factors of disordered C12 and C13 atoms refined at 0.718 (11) for major position and 0.282 (11) for minor position. The anisotropic displacement parameters of C3 and C4 were restrained with DELU in the final cycles of refinement.

### Crystal data

**Table:**

C_24_H_17_NO_4_S	*F*(000) = 1728
*M**_r_* = 415.45	*D*_x_ = 1.418 Mg m^−^^3^
Orthorhombic, *P**b**c**a*	Mo *K*α radiation, λ = 0.71073 Å
Hall symbol: -P 2ac 2ab	Cell parameters from 5557 reflections
*a* = 13.189 (5) Å	θ = 2.3–27.8°
*b* = 16.363 (6) Å	µ = 0.20 mm^−^^1^
*c* = 18.039 (5) Å	*T* = 295 K
*V* = 3893 (2) Å^3^	Block, colourless
*Z* = 8	0.26 × 0.22 × 0.20 mm

### Data collection

**Table:**

Bruker Kappa APEXII diffractometer	4813 independent reflections
Radiation source: fine-focus sealed tube	3469 reflections with *I* > 2σ(*I*)
graphite	*R*_int_ = 0.032
ω and φ scans	θ_max_ = 28.3°, θ_min_ = 2.3°
Absorption correction: multi-scan (*SADABS*; Sheldrick, 1996)	*h* = −17→17
*T*_min_ = 0.950, *T*_max_ = 0.961	*k* = −16→21
19551 measured reflections	*l* = −23→17

### Refinement

**Table:**

Refinement on *F*^2^	Primary atom site location: structure-invariant direct methods
Least-squares matrix: full	Secondary atom site location: difference Fourier map
*R*[*F*^2^ > 2σ(*F*^2^)] = 0.041	Hydrogen site location: inferred from neighbouring sites
*wR*(*F*^2^) = 0.124	H-atom parameters constrained
*S* = 1.03	*w* = 1/[σ^2^(*F*_o_^2^) + (0.0637*P*)^2^ + 0.6676*P*] where *P* = (*F*_o_^2^ + 2*F*_c_^2^)/3
4813 reflections	(Δ/σ)_max_ < 0.001
290 parameters	Δρ_max_ = 0.28 e Å^−^^3^
1 restraint	Δρ_min_ = −0.36 e Å^−^^3^

### Fractional atomic coordinates and isotropic or equivalent isotropic displacement parameters (Å^2^)

**Table:**

	*x*	*y*	*z*	*U*_iso_*/*U*_eq_	Occ. (<1)
S1	0.75886 (3)	0.20738 (3)	0.50676 (2)	0.04410 (14)	
O1	0.68946 (12)	0.14774 (9)	0.48039 (8)	0.0645 (4)	
O2	0.86322 (10)	0.20175 (9)	0.48726 (7)	0.0609 (4)	
O3	1.18355 (9)	0.45554 (8)	0.71672 (8)	0.0547 (3)	
O4	1.10235 (10)	0.45229 (9)	0.86231 (7)	0.0614 (4)	
N1	0.75360 (10)	0.20284 (8)	0.59811 (8)	0.0412 (3)	
C1	0.71502 (13)	0.30481 (11)	0.48204 (9)	0.0429 (4)	
C2	0.61251 (14)	0.32135 (13)	0.48674 (10)	0.0541 (5)	
H2	0.5677	0.2819	0.5041	0.065*	
C3	0.57779 (19)	0.39686 (16)	0.46545 (12)	0.0728 (6)	
H3	0.5089	0.4087	0.4678	0.087*	
C4	0.6445 (3)	0.45467 (16)	0.44081 (14)	0.0850 (7)	
H4	0.6205	0.5057	0.4266	0.102*	
C5	0.7463 (2)	0.43862 (16)	0.43669 (15)	0.0840 (8)	
H5	0.7908	0.4788	0.4200	0.101*	
C6	0.78291 (17)	0.36299 (14)	0.45719 (12)	0.0636 (6)	
H6	0.8518	0.3514	0.4544	0.076*	
C7	0.81876 (11)	0.24936 (10)	0.64551 (8)	0.0348 (3)	
C8	0.90603 (11)	0.29052 (10)	0.62968 (8)	0.0379 (3)	
H8	0.9320	0.2914	0.5818	0.046*	
C9	0.95611 (11)	0.33194 (9)	0.68816 (8)	0.0343 (3)	
C10	1.04706 (12)	0.37570 (10)	0.67599 (9)	0.0397 (4)	
H10	1.0742	0.3780	0.6284	0.048*	
C11	1.09563 (11)	0.41443 (10)	0.73221 (9)	0.0396 (4)	
C12	1.2233 (3)	0.5044 (3)	0.7720 (3)	0.0511 (10)	0.718 (11)
H12A	1.1901	0.5573	0.7710	0.061*	0.718 (11)
H12B	1.2950	0.5128	0.7631	0.061*	0.718 (11)
C12A	1.2432 (9)	0.4744 (9)	0.7890 (7)	0.054 (3)	0.282 (11)
H12C	1.2765	0.4249	0.8057	0.064*	0.282 (11)
H12D	1.2956	0.5143	0.7780	0.064*	0.282 (11)
C13	1.2089 (3)	0.4664 (3)	0.8461 (2)	0.0523 (11)	0.718 (11)
H13A	1.2450	0.4147	0.8478	0.063*	0.718 (11)
H13B	1.2375	0.5018	0.8838	0.063*	0.718 (11)
C13A	1.1788 (7)	0.5065 (9)	0.8505 (5)	0.054 (3)	0.282 (11)
H13C	1.2189	0.5128	0.8951	0.065*	0.282 (11)
H13D	1.1512	0.5595	0.8371	0.065*	0.282 (11)
C14	1.05574 (12)	0.41171 (10)	0.80512 (9)	0.0424 (4)	
C15	0.96920 (13)	0.36957 (10)	0.81897 (9)	0.0428 (4)	
H15	0.9442	0.3674	0.8671	0.051*	
C16	0.91622 (11)	0.32886 (10)	0.76166 (8)	0.0355 (3)	
C17	0.82565 (11)	0.28589 (10)	0.77520 (9)	0.0385 (3)	
H17	0.7989	0.2837	0.8229	0.046*	
C18	0.77697 (11)	0.24729 (9)	0.71804 (8)	0.0345 (3)	
C19	0.68351 (11)	0.20045 (9)	0.71441 (9)	0.0369 (3)	
C20	0.61522 (12)	0.17749 (11)	0.76943 (10)	0.0456 (4)	
H20	0.6249	0.1938	0.8183	0.055*	
C21	0.53295 (14)	0.13020 (12)	0.75009 (12)	0.0569 (5)	
H21	0.4869	0.1139	0.7862	0.068*	
C22	0.51865 (14)	0.10687 (13)	0.67716 (13)	0.0599 (5)	
H22	0.4619	0.0759	0.6650	0.072*	
C23	0.58604 (14)	0.12802 (12)	0.62164 (11)	0.0540 (5)	
H23	0.5763	0.1112	0.5729	0.065*	
C24	0.66883 (12)	0.17543 (10)	0.64165 (9)	0.0406 (4)	

### Atomic displacement parameters (Å^2^)

**Table:**

	*U*^11^	*U*^22^	*U*^33^	*U*^12^	*U*^13^	*U*^23^
S1	0.0514 (2)	0.0484 (3)	0.0325 (2)	0.00302 (18)	−0.00712 (17)	−0.00640 (17)
O1	0.0849 (10)	0.0577 (9)	0.0509 (8)	−0.0113 (7)	−0.0220 (7)	−0.0092 (7)
O2	0.0564 (8)	0.0862 (11)	0.0400 (7)	0.0196 (7)	0.0003 (6)	−0.0129 (7)
O3	0.0468 (6)	0.0591 (8)	0.0582 (8)	−0.0183 (6)	0.0014 (6)	0.0065 (6)
O4	0.0638 (8)	0.0722 (9)	0.0484 (8)	−0.0254 (7)	−0.0039 (6)	−0.0135 (7)
N1	0.0462 (7)	0.0441 (8)	0.0334 (7)	−0.0060 (6)	−0.0073 (5)	0.0000 (6)
C1	0.0489 (9)	0.0498 (10)	0.0300 (8)	0.0005 (8)	−0.0015 (7)	0.0029 (7)
C2	0.0510 (10)	0.0658 (12)	0.0456 (10)	0.0058 (9)	0.0012 (8)	0.0040 (9)
C3	0.0784 (14)	0.0827 (16)	0.0572 (13)	0.0317 (11)	0.0006 (11)	0.0054 (11)
C4	0.129 (2)	0.0613 (15)	0.0647 (15)	0.0263 (12)	0.0033 (14)	0.0147 (11)
C5	0.113 (2)	0.0669 (16)	0.0722 (17)	−0.0160 (15)	0.0085 (14)	0.0251 (13)
C6	0.0626 (11)	0.0692 (14)	0.0588 (13)	−0.0094 (10)	0.0055 (10)	0.0160 (10)
C7	0.0390 (7)	0.0341 (8)	0.0313 (8)	0.0031 (6)	−0.0038 (6)	−0.0002 (6)
C8	0.0414 (8)	0.0449 (9)	0.0275 (7)	−0.0010 (7)	0.0033 (6)	0.0006 (7)
C9	0.0365 (7)	0.0359 (8)	0.0304 (8)	0.0018 (6)	0.0033 (6)	0.0012 (6)
C10	0.0427 (8)	0.0435 (9)	0.0329 (8)	−0.0023 (7)	0.0065 (6)	0.0039 (7)
C11	0.0379 (7)	0.0352 (8)	0.0456 (9)	−0.0025 (6)	0.0013 (6)	0.0051 (7)
C12	0.0400 (17)	0.047 (2)	0.066 (3)	−0.0101 (15)	−0.0075 (15)	−0.0009 (18)
C12A	0.053 (5)	0.051 (6)	0.057 (6)	−0.002 (4)	−0.019 (4)	−0.005 (5)
C13	0.0498 (17)	0.049 (2)	0.058 (2)	−0.0055 (16)	−0.0143 (15)	−0.0046 (17)
C13A	0.045 (4)	0.048 (6)	0.070 (5)	−0.003 (4)	−0.010 (3)	−0.007 (4)
C14	0.0490 (9)	0.0400 (9)	0.0383 (9)	−0.0039 (7)	−0.0035 (7)	−0.0036 (7)
C15	0.0494 (9)	0.0476 (10)	0.0314 (8)	−0.0075 (7)	0.0058 (7)	−0.0049 (7)
C16	0.0389 (7)	0.0356 (8)	0.0321 (8)	0.0010 (6)	0.0040 (6)	−0.0014 (6)
C17	0.0427 (8)	0.0420 (9)	0.0308 (8)	−0.0030 (7)	0.0075 (6)	−0.0014 (7)
C18	0.0359 (7)	0.0311 (8)	0.0364 (8)	0.0017 (6)	0.0027 (6)	0.0013 (6)
C19	0.0357 (7)	0.0312 (8)	0.0438 (9)	0.0023 (6)	−0.0007 (6)	0.0037 (7)
C20	0.0417 (8)	0.0439 (9)	0.0513 (10)	−0.0004 (7)	0.0059 (7)	0.0032 (8)
C21	0.0424 (9)	0.0562 (12)	0.0721 (13)	−0.0059 (8)	0.0055 (9)	0.0094 (10)
C22	0.0437 (9)	0.0555 (12)	0.0805 (15)	−0.0141 (8)	−0.0105 (9)	0.0095 (10)
C23	0.0534 (10)	0.0525 (11)	0.0561 (11)	−0.0090 (8)	−0.0173 (9)	0.0040 (9)
C24	0.0407 (8)	0.0345 (8)	0.0466 (10)	0.0011 (7)	−0.0075 (7)	0.0063 (7)

### Geometric parameters (Å, °)

**Table:**

S1—O1	1.4201 (14)	C10—H10	0.9300
S1—O2	1.4237 (15)	C11—C14	1.417 (2)
S1—N1	1.6509 (15)	C12—C13	1.486 (7)
S1—C1	1.7535 (19)	C12—H12A	0.9700
O3—C11	1.3695 (19)	C12—H12B	0.9700
O3—C12	1.381 (4)	C12A—C13A	1.492 (19)
O3—C12A	1.555 (11)	C12A—H12C	0.9700
O4—C13A	1.360 (8)	C12A—H12D	0.9700
O4—C14	1.372 (2)	C13—H13A	0.9700
O4—C13	1.454 (4)	C13—H13B	0.9700
N1—C7	1.4315 (19)	C13A—H13C	0.9700
N1—C24	1.438 (2)	C13A—H13D	0.9700
C1—C2	1.381 (2)	C14—C15	1.357 (2)
C1—C6	1.382 (3)	C15—C16	1.414 (2)
C2—C3	1.373 (3)	C15—H15	0.9300
C2—H2	0.9300	C16—C17	1.407 (2)
C3—C4	1.367 (4)	C17—C18	1.369 (2)
C3—H3	0.9300	C17—H17	0.9300
C4—C5	1.370 (4)	C18—C19	1.453 (2)
C4—H4	0.9300	C19—C24	1.388 (2)
C5—C6	1.379 (3)	C19—C20	1.392 (2)
C5—H5	0.9300	C20—C21	1.378 (3)
C6—H6	0.9300	C20—H20	0.9300
C7—C8	1.364 (2)	C21—C22	1.383 (3)
C7—C18	1.420 (2)	C21—H21	0.9300
C8—C9	1.417 (2)	C22—C23	1.383 (3)
C8—H8	0.9300	C22—H22	0.9300
C9—C10	1.414 (2)	C23—C24	1.387 (2)
C9—C16	1.427 (2)	C23—H23	0.9300
C10—C11	1.357 (2)		
			
O1—S1—O2	119.73 (9)	C13A—C12A—O3	113.9 (9)
O1—S1—N1	106.04 (8)	C13A—C12A—H12C	108.8
O2—S1—N1	106.52 (7)	O3—C12A—H12C	108.8
O1—S1—C1	109.09 (9)	C13A—C12A—H12D	108.8
O2—S1—C1	108.35 (9)	O3—C12A—H12D	108.8
N1—S1—C1	106.32 (8)	H12C—C12A—H12D	107.7
C11—O3—C12	117.3 (2)	O4—C13—C12	111.7 (4)
C11—O3—C12A	110.8 (5)	O4—C13—H13A	109.3
C13A—O4—C14	122.0 (4)	C12—C13—H13A	109.3
C14—O4—C13	111.0 (2)	O4—C13—H13B	109.3
C7—N1—C24	107.84 (13)	C12—C13—H13B	109.3
C7—N1—S1	123.16 (11)	H13A—C13—H13B	107.9
C24—N1—S1	126.29 (11)	O4—C13A—C12A	108.0 (10)
C2—C1—C6	121.28 (19)	O4—C13A—H13C	110.1
C2—C1—S1	119.03 (15)	C12A—C13A—H13C	110.1
C6—C1—S1	119.69 (15)	O4—C13A—H13D	110.1
C3—C2—C1	119.1 (2)	C12A—C13A—H13D	110.1
C3—C2—H2	120.5	H13C—C13A—H13D	108.4
C1—C2—H2	120.5	C15—C14—O4	118.97 (15)
C4—C3—C2	119.9 (2)	C15—C14—C11	119.94 (15)
C4—C3—H3	120.0	O4—C14—C11	121.08 (15)
C2—C3—H3	120.0	C14—C15—C16	121.36 (15)
C3—C4—C5	121.1 (2)	C14—C15—H15	119.3
C3—C4—H4	119.5	C16—C15—H15	119.3
C5—C4—H4	119.5	C17—C16—C15	121.85 (14)
C4—C5—C6	120.0 (2)	C17—C16—C9	119.45 (14)
C4—C5—H5	120.0	C15—C16—C9	118.70 (14)
C6—C5—H5	120.0	C18—C17—C16	119.86 (14)
C5—C6—C1	118.6 (2)	C18—C17—H17	120.1
C5—C6—H6	120.7	C16—C17—H17	120.1
C1—C6—H6	120.7	C17—C18—C7	120.05 (14)
C8—C7—C18	122.16 (14)	C17—C18—C19	132.47 (14)
C8—C7—N1	130.11 (14)	C7—C18—C19	107.48 (13)
C18—C7—N1	107.73 (13)	C24—C19—C20	120.28 (15)
C7—C8—C9	118.27 (14)	C24—C19—C18	108.45 (14)
C7—C8—H8	120.9	C20—C19—C18	131.24 (15)
C9—C8—H8	120.9	C21—C20—C19	118.73 (18)
C10—C9—C8	121.46 (14)	C21—C20—H20	120.6
C10—C9—C16	118.34 (14)	C19—C20—H20	120.6
C8—C9—C16	120.18 (14)	C20—C21—C22	120.22 (18)
C11—C10—C9	121.40 (15)	C20—C21—H21	119.9
C11—C10—H10	119.3	C22—C21—H21	119.9
C9—C10—H10	119.3	C21—C22—C23	122.16 (17)
C10—C11—O3	118.47 (15)	C21—C22—H22	118.9
C10—C11—C14	120.25 (14)	C23—C22—H22	118.9
O3—C11—C14	121.27 (15)	C22—C23—C24	117.21 (18)
O3—C12—C13	111.0 (4)	C22—C23—H23	121.4
O3—C12—H12A	109.4	C24—C23—H23	121.4
C13—C12—H12A	109.4	C23—C24—C19	121.38 (16)
O3—C12—H12B	109.4	C23—C24—N1	130.11 (16)
C13—C12—H12B	109.4	C19—C24—N1	108.42 (13)
H12A—C12—H12B	108.0		
			
O1—S1—N1—C7	174.92 (13)	O3—C12A—C13A—O4	55.0 (17)
O2—S1—N1—C7	46.36 (15)	C13A—O4—C14—C15	−168.7 (8)
C1—S1—N1—C7	−69.05 (14)	C13—O4—C14—C15	158.0 (3)
O1—S1—N1—C24	−26.04 (16)	C13A—O4—C14—C11	10.2 (8)
O2—S1—N1—C24	−154.61 (14)	C13—O4—C14—C11	−23.1 (3)
C1—S1—N1—C24	89.99 (15)	C10—C11—C14—C15	0.6 (3)
O1—S1—C1—C2	39.85 (17)	O3—C11—C14—C15	−179.12 (16)
O2—S1—C1—C2	171.74 (14)	C10—C11—C14—O4	−178.27 (16)
N1—S1—C1—C2	−74.10 (15)	O3—C11—C14—O4	2.0 (3)
O1—S1—C1—C6	−139.10 (16)	O4—C14—C15—C16	177.91 (16)
O2—S1—C1—C6	−7.21 (17)	C11—C14—C15—C16	−1.0 (3)
N1—S1—C1—C6	106.94 (16)	C14—C15—C16—C17	−179.13 (16)
C6—C1—C2—C3	0.7 (3)	C14—C15—C16—C9	0.7 (2)
S1—C1—C2—C3	−178.21 (16)	C10—C9—C16—C17	179.83 (15)
C1—C2—C3—C4	−0.7 (3)	C8—C9—C16—C17	−1.4 (2)
C2—C3—C4—C5	0.2 (4)	C10—C9—C16—C15	0.0 (2)
C3—C4—C5—C6	0.4 (4)	C8—C9—C16—C15	178.78 (15)
C4—C5—C6—C1	−0.3 (4)	C15—C16—C17—C18	−179.92 (15)
C2—C1—C6—C5	−0.2 (3)	C9—C16—C17—C18	0.3 (2)
S1—C1—C6—C5	178.73 (18)	C16—C17—C18—C7	1.1 (2)
C24—N1—C7—C8	−177.15 (16)	C16—C17—C18—C19	−178.74 (16)
S1—N1—C7—C8	−14.8 (2)	C8—C7—C18—C17	−1.5 (2)
C24—N1—C7—C18	2.68 (17)	N1—C7—C18—C17	178.69 (14)
S1—N1—C7—C18	165.04 (11)	C8—C7—C18—C19	178.43 (14)
C18—C7—C8—C9	0.3 (2)	N1—C7—C18—C19	−1.41 (16)
N1—C7—C8—C9	−179.87 (15)	C17—C18—C19—C24	179.46 (17)
C7—C8—C9—C10	179.83 (15)	C7—C18—C19—C24	−0.42 (17)
C7—C8—C9—C16	1.1 (2)	C17—C18—C19—C20	−2.7 (3)
C8—C9—C10—C11	−179.13 (15)	C7—C18—C19—C20	177.44 (16)
C16—C9—C10—C11	−0.4 (2)	C24—C19—C20—C21	−0.4 (2)
C9—C10—C11—O3	179.82 (14)	C18—C19—C20—C21	−178.10 (16)
C9—C10—C11—C14	0.1 (2)	C19—C20—C21—C22	−0.5 (3)
C12—O3—C11—C10	170.5 (3)	C20—C21—C22—C23	1.4 (3)
C12A—O3—C11—C10	−164.0 (6)	C21—C22—C23—C24	−1.1 (3)
C12—O3—C11—C14	−9.7 (3)	C22—C23—C24—C19	0.1 (3)
C12A—O3—C11—C14	15.8 (6)	C22—C23—C24—N1	176.05 (17)
C11—O3—C12—C13	37.2 (6)	C20—C19—C24—C23	0.7 (2)
C12A—O3—C12—C13	−42.9 (14)	C18—C19—C24—C23	178.81 (15)
C11—O3—C12A—C13A	−44.9 (14)	C20—C19—C24—N1	−176.06 (14)
C12—O3—C12A—C13A	65.6 (16)	C18—C19—C24—N1	2.09 (17)
C13A—O4—C13—C12	−66.6 (9)	C7—N1—C24—C23	−179.30 (17)
C14—O4—C13—C12	50.7 (5)	S1—N1—C24—C23	19.0 (3)
O3—C12—C13—O4	−59.1 (7)	C7—N1—C24—C19	−2.97 (17)
C14—O4—C13A—C12A	−37.8 (15)	S1—N1—C24—C19	−164.63 (12)
C13—O4—C13A—C12A	40.2 (10)		

### Hydrogen-bond geometry (Å, °)

**Table:**

Cg1, Cg4, Cg5 and Cg7 are the centroids of the N1/C7/C18/C19/C24, C1–C6, C7–C9/C16–C18 and C19–C24 rings, respectively.

**Table:**

*D*—H···*A*	*D*—H	H···*A*	*D*···*A*	*D*—H···*A*
C6—H6···O2	0.93	2.52	2.894 (3)	104
C8—H8···O2	0.93	2.42	3.005 (2)	120
C23—H23···O1	0.93	2.32	2.908 (3)	121
C10—H10···O1^i^	0.93	2.52	3.411 (2)	161
C12—H12A···Cg1^ii^	0.97	2.98	3.670 (5)	129
C12—H12B···Cg7^ii^	0.97	2.78	3.411 (5)	124
C13—H13A···Cg5^iii^	0.97	2.77	3.680 (5)	157
C20—H20···Cg4^iv^	0.93	2.94	3.689 (2)	138
C12A—H12C···Cg5^iii^	0.97	2.53	3.446 (14)	158
C12A—H12D···Cg7^ii^	0.97	2.69	3.585 (14)	153
C13A—H13D···Cg1^ii^	0.97	2.92	3.585 (14)	127

Symmetry codes: (i) *x*+1/2, −*y*+1/2, −*z*+1; (ii) −*x*+2, *y*+1/2, −*z*+3/2; (iii) *x*−1/2, *y*, −*z*+1/2; (iv) *x*, −*y*−1/2, *z*−1/2.
